# New Insights into Rate Control: Time in Target Range of Resting Heart Rate and Major Adverse Outcomes in Atrial Fibrillation

**DOI:** 10.5334/gh.1251

**Published:** 2024-01-11

**Authors:** Yuhui Lai, Xingfeng Xu, Shaozhao Zhang, Rihua Huang, Yiquan Huang, Xiangbin Zhong, Zhenyu Xiong, Yifen Lin, Huimin Zhou, Yue Guo, Xinxue Liao, Yuedong Ma, Xiaodong Zhuang

**Affiliations:** 1 Cardiology department, first affiliated hospital of Sun Yat-Sen University, CN; 2 NHC Key Laboratory of Assisted Circulation, Sun Yat-sen University, CN

**Keywords:** resting heart rate, rate control, time in target range, atrial fibrillation

## Abstract

**Background:**

Few studies have examined the relationship between the fluctuation of heart rate control over time and cardiovascular outcomes in patients with atrial fibrillation. Our study sought to evaluate the independent association between time in target range (TIR) of resting heart rate and cardiovascular outcomes in the AFFIRM (Atrial Fibrillation Follow-Up Investigation of Rhythm Management) study.

**Methods:**

Target range of resting heart was defined as less than 80 beats per minute (bpm) for both rate and rhythm control groups. Time in target range was estimated over the first 8 months of follow-up using Rosendaal interpolation method. The association between TIR of resting heart rate and cardiovascular outcomes was estimated using adjusted Cox proportional hazards regression models.

**Results:**

Time in target range of resting heart rate (months 0 through 8) was 71 ± 34% in the rate control group and 83 ± 27% in the rhythm control group. Each 1-SD increase in TIR of resting heart rate was significantly associated with lower risk of major adverse cardiovascular events after full adjustment for demographics, medical history and history of prior heart surgery, as well as all-cause mortality.

**Conclusions:**

Time in target range of resting heart rate independently predicts the risk of cardiovascular outcomes in patients with atrial fibrillation. Long-term maintenance of heart rate on target is of great importance for patients with atrial fibrillation.

## Introduction

Atrial fibrillation (AF) is the most common cardiac arrhythmia worldwide and is associated with significant morbidity and mortality [1]. It may cause symptoms of and is related to stroke and heart failure. Control of the heart rate is central to atrial fibrillation management, even for patients who ultimately require control of the rhythm [2], which can reduce symptoms, improve hemodynamics, and prevent adverse cardiovascular outcomes. Higher heart rate is associated with an increased risk of cardiovascular disease (CVD) and cardiovascular mortality [3,4]. As an important sign, the level of heart rate control is often determined using mean heart rate or a single measurement within the observation period. It is problematic because heart rate is a time-dependent sign which fluctuates over time; therefore, the traditional methods may not adequately reflect the control level of resting heart rate over a period of time.

The traditional measure of heart rate control only accounts for the overall level of a patient’s resting heart rate, lacking the information of longitudinal variations within and out of target range. The mean of multiple resting heart rate measurements over a period of time may be within target range, but the time of reaching the expected level remains unknown. Herein, we chose time in target range (TIR) of resting heart rate to evaluate the level of heart rate control more appropriately.

The association between heart rate control over a period of time and subsequent clinical outcomes has not been well described. Our study aimed to: (1) describe the levels of heart rate control in patients with atrial fibrillation using rate control and rhythm control strategies; (2) assess the relationship between TIR of resting heart rate and clinical outcomes among adults with atrial fibrillation and to determine whether TIR predicts adverse outcomes independent of mean resting heart rate and heart rate variability.

## Methods

### Population

The AFFIRM trial was a prospective randomized trial, conducted by the US National Heart, Lung, and Blood Institute. The study protocol and the principal trial results have been described in detail elsewhere [5]. The mean duration of follow-up was 3.5 years.

Institutional review board for every institution approved study protocol. All patients entered the study after written informed consent. The study was performed according to the European Union Note for Guidance on Good Clinical Practice CPMP/ECH/135/95 and the Declaration of Helsinki [6]. Our study was a substudy of the AFFIRM trial, which was approved by the Institutional Review Board at Sun Yat-sen University in the Committee on Human Research.

This substudy considered all AFFIRM study patients in the rate- and rhythm-control arms who had at least 4 resting heart rate measurements documented during the exposure period (months 0 through 8). Participants were excluded from certain time-to-event analyses if they experienced that event during the exposure period or were lost to follow-up. For example, participants who experienced a nonfatal arrhythmia during the exposure period were excluded from time to first major adverse cardiovascular event analyses. We evaluated the association of the time of resting heart rate in target range with major adverse cardiovascular events and all-cause mortality.

### Rhythm-control Strategy

In the rhythm-control group, the antiarrhythmic drug used was chosen by the treating physician. Attempts to maintain sinus rhythm could include cardioversion as necessary. The following drugs were acceptable for use, according to the protocol: amiodarone, disopyramide, flecainide, moricizine, procainamide, propafenone, quinidine, sotalol and combinations of these drugs. When dofetilide became available, it could also be used. Specific guidelines for the use of antiarrhythmic drugs were imposed.

### Rate-control Strategy

The therapeutic target in this group was heart rate control. Drugs that were acceptable in the protocol for this purpose were beta-blockers, calcium-channel blockers (verapamil and diltiazem), digoxin and combinations of these drugs. Heart rate control during atrial fibrillation was assessed both at rest and during activity, which usually consisted of a six-minute walk. The goal was a heart rate not higher than 80 beats per minute at rest and not higher than 110 beats per minute during the six-minute walk test.

### Other Therapeutic Considerations

After standard approaches to treatment were exhausted, but not before the failure of at least two trials of either a rhythm-control drug or a rate-control drug, patients could be considered for nonpharmacologic therapy, such as radio-frequency ablation, a maze procedure, and pacing techniques, as appropriate to their randomized strategy.

### Resting Heart Rate Control Measures

Resting heart rate was assessed in all patients at baseline and at subsequent two-month visits. Heart rate at rest was obtained by apical auscultation for 1 minute after the patient had been sitting quietly for ≥5 minutes.

We defined a longitudinal measure of resting heart rate control during months 0 through 8 (0, 2, 4, 8 months). Time in target range of resting heart rate was calculated according to Rosendaal interpolation method [7]. This method adds each patient’s time within the target range and divides it by the total time of observation. This assumes that between-measurement heart rate varies linearly.

Considering the sufficient control of resting heart rate in this population, the upper limit of target range was defined as 80 bpm for both rate and rhythm control groups, which were selected to further explore the target of heart rate control [8]. Mean resting heart rate and resting heart rate standard deviation (SD) were calculated with all resting heart rate measurements during months 0–8.

### Treatment During Follow-up

Patients assigned to the rhythm-control strategy in the AFFIRM study were prescribed antiarrhythmic drugs commonly used to establish and/or maintain sinus rhythm. Patients assigned to the rate-control strategy received digoxin, non-dihydropyridine calcium antagonists and/or beta-blockers [9]. The treating physician prescribed rhythm- and rate-control drugs based on prespecified guidelines [9,10,11,12]. Adequacy of rhythm-and rate-control was determined by history, physical examination and an ECG rhythm strip performed at least at each four-month visit. Rate- and rhythm-control drugs were adjusted as necessary. Cardioversion was performed as needed in the rhythm-control group. If adequate rhythm or rate control could not be achieved with at least two medications given separately, the treating physician could proceed with an innovative therapy.

### Cardiovascular Outcomes

Outcomes included major adverse cardiovascular events (cardiovascular death, arrhythmia including torsades de pointes VT, sustained ventricular tachycardia and resuscitated cardiac arrest: VF, VT, EMD, brady and others, ischemic stroke, major bleeding, systemic or pulmonary embolism, and myocardial infarction) and all-cause mortality. Structured interviews were performed within 24 hours of learning about a patient’s death or within one week of learning about other events. Relevant data were gathered according to a standard protocol, and events were adjudicated by an independent committee blinded to treatment allocation.

### Statistical Analysis

Categorical data were summarized as counts and percentages, and continuous data were expressed as either mean ± SD or median (interquartile range). Continuous baseline data were compared across the 3 TIR groups (0%–50%, >50%–75%, >75%–100%) using the analysis of variance test, and categorical baseline data were compared using the chi-square test.

The associations between TIR of resting heart rate and the first occurrence of an outcome were estimated using hazard ratios (HRs) and 95% confidence intervals (CIs) derived from Cox proportional regression models. In the first model, we adjusted for age, gender, minority, smoking status and randomized treatment group. In the second model, we further adjusted for medical history, including hypertension, diabetes mellitus, coronary artery diseases and congestive heart failure. In the final model, we further adjusted for history of coronary artery bypass graft, interventional procedures and pacemaker implantation. HRs for each TIR exposure were estimated per 1-SD increase.

The analysis of the association between TIR of resting heart rate and cardiovascular outcomes was performed in the complete study cohort. Multiplicative interaction terms were used to assess an interaction between the randomization arm and association of TIR with cardiovascular outcomes.

We also performed sensitivity analyses in patients who had four heart rate measurements all during sinus rhythm or atrial fibrillation to explore whether the association between TIR of resting heart rate and cardiovascular outcomes was consistent on different rhythm status.

A p value <0.05 was considered statistically significant. All analyses were performed using R (version 4.1.0; R Foundation for Statistical Computing).

## Results

### Overall Study Population Characteristics

After excluding 612 participants with less than 4 resting heart rate measurement and 142 participants with missing covariate data, the final study cohort included 3,306 participants (of total population of 4,061 AFFIRM participants). Baseline characteristics are shown in Table 1. The mean age of the study population was 70 ± 8 years; 1,287 (39%) were women, and 2,976 (90%) were Caucasians (Table 1).

**Table 1:** Characteristics of Participants According to Time in Target Range of Resting Heart Rate.


VARIABLES MEAN ± SD OR n (%)	OVERALL (n = 3306)	TIR GROUP			

		0%~50% (n = 662)	>50~75% (n = 454)	>75~100% (n = 2190)	P VALUE

Age	70 ± 8	69 ± 8	69 ± 8	70 ± 8	0.046

Gender					0.632

Male	2019 (61%)	398 (60%)	271 (60%)	1350 (62%)	

Female	1287 (39%)	264 (40%)	183 (40%)	840 (38%)	

Minority					0.861

Caucasian	2976 (90%)	599 (91%)	410 (90%)	1967 (90%)	

Non-Caucasian	330 (10%)	63 (10%)	44 (10%)	223 (10%)	

Smoking					<0.001

Non-smoker	2927 (90%)	557 (84%)	397 (87%)	1973 (90%)	

Smoking(within 2 years)	379 (12%)	105 (16%)	57 (13%)	217 (10%)	

Randomized treatment group					<0.001

Rate control	1653 (50%)	435 (66%)	252 (56%)	966 (44%)	

Rhythm control	1653 (50%)	227 (34%)	202 (45%)	1224 (56%)	

Hypertension	2346 (71%)	466 (70%)	317 (70%)	1563 (71%)	0.753

Diabetes mellitus	645 (20%)	149 (23%)	107 (24%)	389 (18%)	0.002

Coronary artery disease	1234 (37%)	234 (35%)	165 (36%)	835 (38%)	0.387

Pacemaker implantation	194 (6%)	32 (5%)	28 (6%)	134 (6%)	0.448

Coronary artery bypass graft	407 (12%)	72 (11%)	56 (12%)	279 (13%)	0.441

Interventional procedure	282 (9%)	45 (7%)	30 (7%)	207 (10%)	0.029

TIR, %	77 ± 31	22 ± 17	64 ± 7	96 ± 6	<0.001

Mean RHR	71 ± 10	84 ± 6	77 ± 4	65 ± 7	<0.001


### Time in target range, mean resting heart rate and resting heart rate SD

Resting heart rate was assessed in all patients at baseline and subsequent 2-month visits. We defined a longitudinal measure of resting heart rate control during months 0 through 8 (0, 2, 4, 8 months). Time in target range (months 0 through 8) was 71 ± 34% in the rate control group and 83 ± 27% in the rhythm control group. The mean resting heart rate (months 0 through 8) was 73 ± 10 bpm in the rate control group and 69 ± 9 bpm in the rhythm control group. The resting heart rate SD (months 0 through 8) was 8 ± 5 bpm in the rate control group and 8 ± 5 bpm in the rhythm control group.

Participants with a TIR of 0% to 50% were somewhat more likely to be smoking within the past 2 years than participants with a TIR of >75% to 100% (Table 1). There was also a higher prevalence of diabetes, as well as a lower prevalence of Interventional procedure history in participants with a TIR of 0% to 50% compared to participants with a TIR of >75% to 100%. Most of the participants with a TIR of >75% to 100% belonged to the rhythm control group while most of the participants with a TIR of 0% to 50% belonged to the rate control group. The proportions of other demographic variables (age, gender and minority) and other medical histories did not differ across TIR groups.

### Associations of Time in Target Range and Cardiovascular Outcomes

The outcome of the first major adverse cardiovascular event occurred in 675 participants over a median follow-up of 3.6 years. Each 1-SD increase in TIR associated significantly with a decreased risk of first major adverse cardiovascular event after adjustment for age, gender, minority, randomized treatment group and history of smoking (HR: 0.83; 95% CI: 0.77 to 0.89; p < 0.001) and after further adjustment for past medical history of hypertension, diabetes mellitus, coronary artery disease and heart failure (HR: 0.84; 95% CI: 0.78 to 0.90; p < 0.001). The association remained significant after full adjustment for demographics, medical history and history of prior heart surgery (HR: 0.84; 95% CI: 0.78 to 0.90; p < 0.001). In fully adjusted models, TIR was also significantly associated with all-cause mortality (HR: 0.82; 95% CI: 0.75 to 0.90; p < 0.001).

The relationships among TIR with major adverse cardiovascular events and all-cause mortality did not significantly differ across the rate and rhythm treatment arms (p interaction >0.2 for all). In fully adjusted models, TIR associated with first major adverse cardiovascular event in the rate (HR: 0.84; 95% CI: 0.76 to 0.92; p < 0.001) and rhythm (HR: 0.84; 95% CI: 0.75 to 0.94; p = 0.002) control groups.

**Figure 1:**
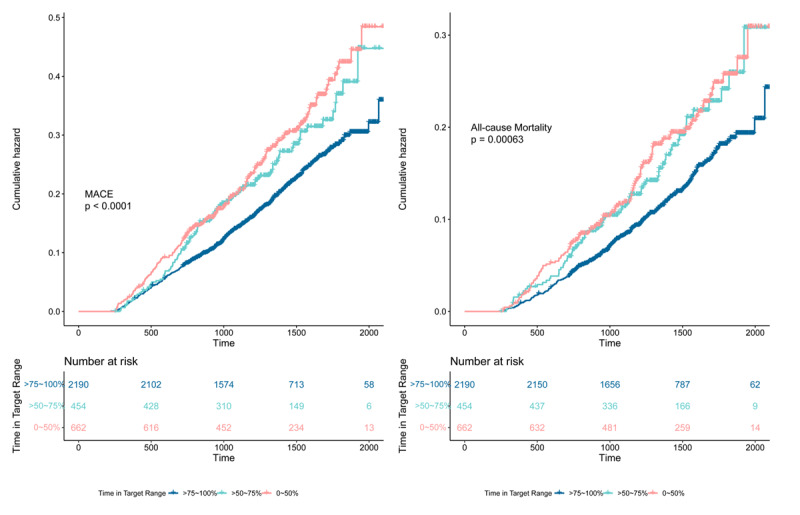
The associations of time in target range groups and cardiovascular outcomes.

In the final cox regression model compared with patients with a TIR of >75% to 100%, those with a TIR of 0% to 50% had a significant association with first major adverse cardiovascular event (odds ratio, 1.56; 95% confidence interval [CI], 1.30–1.88) and all-cause mortality (odds ratio, 1.59; 95% confidence interval [CI], 1.26–2.00), patients with a TIR of >50% to 75% were also significantly associated with first major adverse cardiovascular event (odds ratio, 1.31; 95% confidence interval [CI], 1.06–1.63) and all-cause mortality (odds ratio, 1.38; 95% confidence interval [CI], 1.06–1.80) (Table 2) (Figure 1).

**Table 2:** Risk of Cardiovascular Outcomes for Time in Target Range of Resting Heart Rate.


TIR	MODEL 1		MODEL 2		MODEL 3	
		
	HR (95% CI)	p VALUE	HR (95% CI)	p VALUE	HR (95% CI)	p VALUE

Major adverse cardiovascular events						

0%~50%	1.59(1.32,1.91)	<0.001	1.56(1.30,1.88)	<0.001	1.56(1.30,1.88)	<0.001

>50%~75%	1.40(1.13,1.73)	0.002	1.32(1.07,1.65)	0.011	1.31(1.06,1.63)	0.014

>75%~100%	1.00(Reference)	…	1.00(Reference)	…	1.00(Reference)	…

Per 1 SD	0.83(0.77,0.89)	<0.001	0.84(0.78,0.90)	<0.001	0.84(0.78,0.90)	<0.001

All-cause Mortality						

0%~50%	1.62(1.29,2.03)	<0.001	1.59(1.26,2.00)	<0.001	1.59(1.26,2.00)	<0.001

>50%~75%	1.50(1.15,1.95)	0.003	1.40(1.07,1.83)	0.013	1.38(1.06,1.80)	0.018

>75%~100%	1.00(Reference)	…	1.00(Reference)	…	1.00(Reference)	…

Per 1 SD	0.81(0.75,0.89)	<0.001	0.82(0.75,0.90)	<0.001	0.82(0.75,0.90)	<0.001


HR = hazard ratio. Model 1: adjusted for age, gender, minority, randomization to rate vs. rhythm control strategies and history of smoking; Model 2: further adjusted for past medical history of hypertension, diabetes mellitus, coronary artery disease and heart failure status by NYHA class symptoms; Model 3: further adjusted for history of coronary artery bypass graft, interventional procedure and pacemaker implantation.

### Sensitivity Analyses

**Figure 2:**
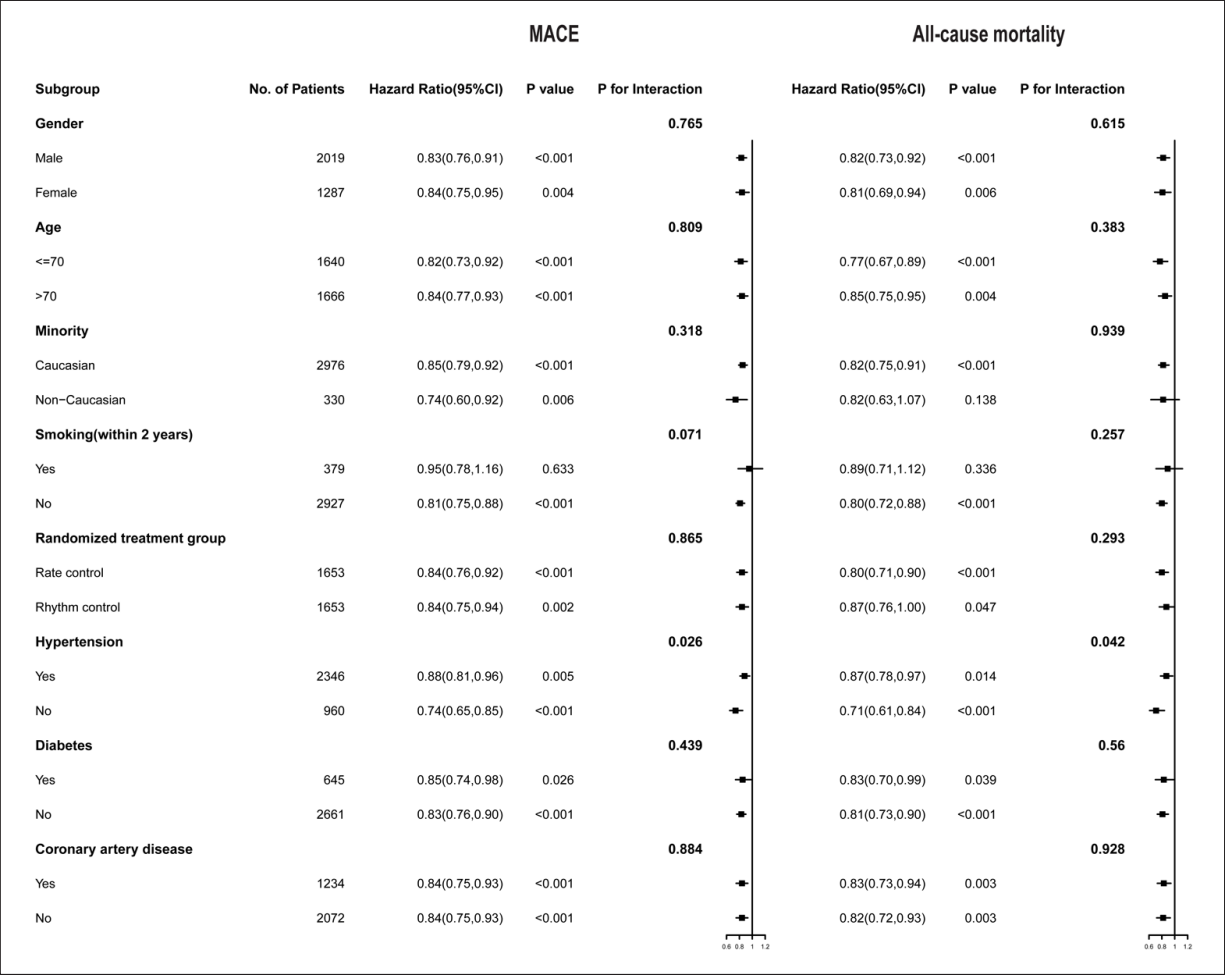
Hazard ratio for cardiovascular outcomes in prespecified subgroups.

In adjusted analyses across key subgroups of interest, when stratified by age (aged ≤70 years or >70 years), minority, smoking status, randomized treatment group and history of diabetes and coronary artery disease, a consistent pattern of association between greater TIR and lower risk of first major adverse cardiovascular event and all-cause mortality was observed. Meanwhile, the association was stronger in patients without hypertension when stratified by the history of hypertension (Figure 2). The interaction between TIR with hypertension was statistically significant (P for interaction <0.05).

In sensitivity analyses, we analyzed whether the association would change if only participants who had four heart rate measurements all during sinus rhythm or atrial fibrillation were selected.

In the sensitivity analysis that excluded participants who had heart rate measurements under AF status, the results did not change substantially. In comparison with the highest TIR group (>75% to 100%), those with a TIR of 0% to 50% had a significant association with first major adverse cardiovascular event (odds ratio, 1.59; 95% confidence interval [CI], 1.10–2.30) and all-cause mortality (odds ratio, 1.90; 95% confidence interval [CI], 1.22–2.96) (Table S1). Each 1-SD increase in TIR associated significantly with a decreased risk of first major adverse cardiovascular event after full adjustment (HR: 0.84; 95% CI: 0.78 to 0.90); p < 0.001). Time in target range was also significantly associated with all-cause mortality (HR: 0.82; 95% CI: 0.75 to 0.90; p < 0.001) (Table 3).

**Table 3:** Associations of Time in Target Range of Resting Heart Rate and Cardiovascular Outcomes With and Without Adjustment for Mean Resting Heart Rate and Resting Heart Rate SD.


OUTCOME	FULLY ADJUSTED^†^		FULLY ADJUSTED PLUS MEAN RHR		FULLY ADJUSTED PLUS RHR SD	
		
	HR* (95% CI)	p VALUE	HR* (95% CI)	p VALUE	HR* (95% CI)	p VALUE

Major adverse cardiovascular events	0.84(0.78,0.90)	<0.001	0.92(0.80,1.05)	0.216	0.83(0.77,0.90)	<0.001

All-cause mortality	0.82(0.75,0.90)	<0.001	0.92(0.78,1.09)	0.348	0.82(0.75,0.90)	<0.001


*HR per 1-SD increase in time in target range of resting heart rate. ^†^Fully adjusted for age, gender, minority, randomization to rate vs. rhythm control strategies, history of smoking, past medical history of hypertension, diabetes mellitus, coronary artery disease and heart failure status by NYHA class symptoms, coronary artery bypass graft, interventional procedure and pacemaker implantation.

In the sensitivity analysis of participants who had four heart rate measurements all during atrial fibrillation, TIR did not associate significantly with first major adverse cardiovascular event (HR: 1.03; 95% CI: 0.87 to 1.22; p = 0.724) and all-cause mortality (HR: 0.99; 95% CI: 0.81 to 1.22; p = 0.949) (Table S2).

## Discussion

**Figure 3:**
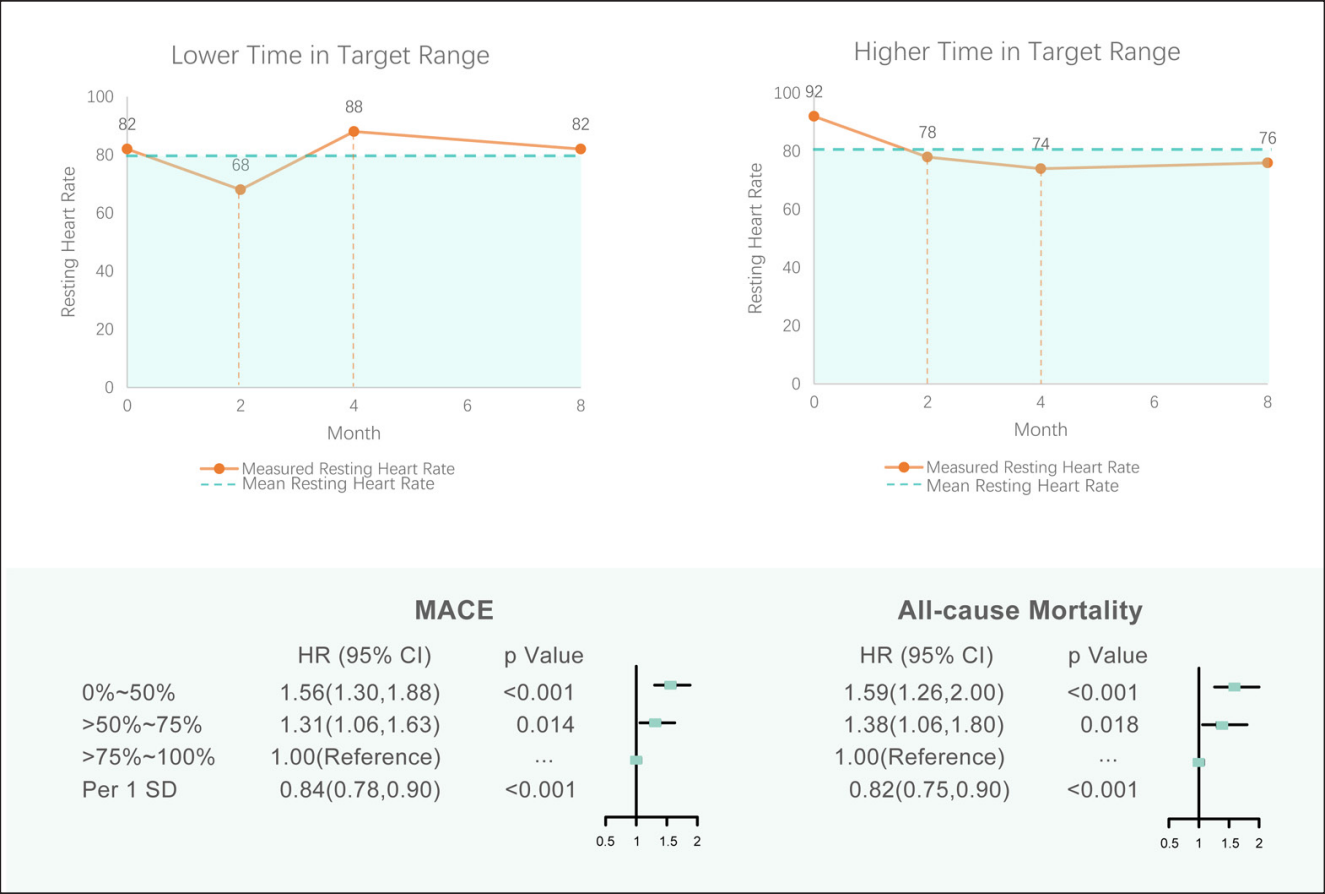
Graphical abstract. Time in target range of resting heart rate as a predictor of cardiovascular outcomes.

Across endpoints and patient populations, lower time with resting heart rate within target range was associated with worse outcomes, including all-cause mortality, as well as major adverse cardiovascular events, independent of traditional cardiovascular risk factors (Figure 3). Importantly, there was no interaction between the randomized group and TIR for the endpoints of major adverse cardiovascular events and all-cause mortality. Outcomes did not differ among pharmacological strategies, indicating that the maintenance of lower heart rate links with better outcomes in both rate and rhythm control groups.

To minimize the effect of rhythm status, we analyzed whether the association would change if only participants who had four heart rate measurements all during sinus rhythm or atrial fibrillation were selected. In the analysis of patients with four measurements all during sinus rhythm, the association between TIR and cardiovascular outcomes did not change. The results confirmed that higher TIR was associated with better cardiovascular outcomes in patients with sinus rhythm. However, in the analysis of patients with four measurements all during AF, the association did not remain significant. Though the result may be brought out by the small sample size (655 patients), it indicates that the AF status should be controlled before we pay close attention to time in target range of resting heart rate.

The main findings of our study do not share opinions with previous studies on the comparison of lenient versus strict rate control in patients with atrial fibrillation. The 2020 ESC Guidelines for the diagnosis and management of atrial fibrillation suggest lenient rate control (heart rate target <110 bpm) rather than strict rate control (heart rate target <80 bpm at rest and <110 bpm at moderate exercise) [13], which is predominantly based on the RACE (Race Control Efficacy in Permanent Atrial Fibrillation) II RCT [14,15]. There was no difference in adverse outcomes between the two rate control strategies in the RACE II trial, similar to an analysis from the AFFIRM and RACE trials [16]. Yet, there were several shortcomings of the conclusions drawn from these trials. The relationship between heart rate and clinical outcomes, particularly mortality, has not been assessed in them. Moreover, it is possible that rapid ventricular rates may take many years to result in cardiac deterioration or other adverse outcomes [17,18], thus there may be a benefit of stricter rate control over decades or more. In the contrast, despite the relatively low heart rates in the population of the present analysis, our data demonstrate a significant association between the time of resting heart rate within a stricter target range (<80 bpm) and adverse outcomes, indicating that stricter heart rate control leads to better clinical outcomes. More importantly, the maintenance of target heart rate needs attention and concern.

Given the divergent treatment guidelines for rate control, our findings have several important clinical implications. Though we cannot definitively identify the optimal heart rate in our study, the association between the time of resting heart rate within a stricter target range (<80 bpm) and adverse outcomes suggests that strict rate control may be associated with superior outcomes. What’s more, at the population-level, these results indicate that mean resting heart rate fails to represent the full spectrum of heart rate-related cardiovascular risk. Time in target range may be a useful tool to characterize the adequacy of rate control, as well as the risk of cardiovascular outcomes. It is of great importance for the judgement of AF patients’ prognosis and subsequent clinical therapy. The linear association of TIR and clinical outcomes suggests maintaining ventricular heart rate within the target range consistently would be a higher level of rate control than single achievement of heart rate target.

## Limitations

The study is retrospective in nature and subject to the limitations inherent to post hoc analyses. We also cannot account for differences in paroxysmal and permanent AF because the type of AF was not available for this analysis. Furthermore, although consistent results were observed across the randomization group and sensitivity analyses, the possibility of residual confounding from unknown or unmeasured variables still cannot be excluded. Also, targeted heart rates for rate control were protocol driven such that observed heart rates in AFFIRM study may not reflect standard clinical practice. Therefore, the impact of very rapid resting heart rates in AF may have been underestimated. Last but not least, the possibility exists that increasing TIR under a lenient target (<110 bpm) may also reduce adverse outcomes. However, a majority of people had a TIR of 100% when the target was 110 bpm, the mean value of TIR was 99% and the median value is 100%, indicating a high degree of heart rate control in this population, making it difficult to achieve an analysis of the TIR under a lenient target.

## Conclusion

Time in target range of resting heart rate independently predicts the risk of cardiovascular outcomes in patients with AF. The current findings underscore that the management of heart rate in patients with AF should not merely focus on the single-point measurement, but also the longitudinal heart rate status. To achieve optimal management in the cardiovascular risk of patients with AF, it may be necessary to control heart rate with a stricter standard over a longer period of time.

## Additional Files

The additional files for this article can be found as follows:

10.5334/gh.1251.s1 Table S1. Risk of Cardiovascular Outcomes for Time in Target Range of Resting Heart Rate in Sensitivity Analysis (Sinus rhythm only).

10.5334/gh.1251.s2 Table S2. Risk of Cardiovascular Outcomes for Time in Target Range of Resting Heart Rate in Sensitivity Analysis (AF only).
