# Supplementary material for: New Insights into Rate Control: Time in Target Range of Resting Heart Rate and Major Adverse Outcomes in Atrial Fibrillation
Source: Glob Heart. 2024 Jan 11;19(1):3. doi: 10.5334/gh.1251 (PMC10786089; doi:10.5334/gh.1251)
Supplement: Table S1. — Risk of Cardiovascular Outcomes for Time in Target Range of Resting Heart Rate in Sensitivity Analysis (Sinus rhythm only). [file gh-19-1-1251-s1.pdf]

TABLE S1 Risk of Cardiovascular Outcomes for Time in Target Range of Resting Heart Rate in Sensitivity Analysis (Sinus rhythm only)

	Model 1		Model 2		Model 3	
TIR	HR (95% CI)	p Value	HR (95% CI)	p Value	HR (95% CI)	p Value
Major adverse cardiovascular events						
0%~50%	1.72(1.19,2.48)	0.004	1.60(1.10,2.32)	0.013	1.59(1.10,2.30)	0.015
>50%~75%	1.21(0.78,1.88)	0.399	1.02(0.65,1.59)	0.947	1.01(0.65,1.58)	0.960
>75%~100%	1.00(Reference)	…	1.00(Reference)	…	1.00(Reference)	…
Per 1 SD	0.83(0.75,0.92)	<0.001	0.86(0.78,0.96)	0.006	0.87(0.78,0.96)	0.008
All-cause Mortality						
0%~50%	2.02(1.31,3.13)	0.002	1.90(1.22,2.95)	0.004	1.90(1.22,2.96)	0.004
>50%~75%	1.37(0.81,2.29)	0.240	1.12(0.66,1.89)	0.684	1.09(0.64,1.85)	0.753
>75%~100%	1.00(Reference)	…	1.00(Reference)	…	1.00(Reference)	…
Per 1 SD	0.79(0.70,0.90)	<0.001	0.82(0.73,0.93)	0.002	0.82(0.73,0.93)	0.002

HR = hazard ratio.

Model 1: adjusted for age, gender, minority, randomization to rate vs. rhythm control strategies and history of smoking;

Model 2: further adjusted for past medical history of hypertension, diabetes mellitus, coronary artery disease and heart failure status by NYHA class symptoms;

Model 3: further adjusted for history of coronary artery bypass graft, interventional procedure and pacemaker implantation
