# Supplementary material for: New Insights into Rate Control: Time in Target Range of Resting Heart Rate and Major Adverse Outcomes in Atrial Fibrillation
Source: Glob Heart. 2024 Jan 11;19(1):3. doi: 10.5334/gh.1251 (PMC10786089; doi:10.5334/gh.1251)
Supplement: Table S2. — Risk of Cardiovascular Outcomes for Time in Target Range of Resting Heart Rate in Sensitivity Analysis (AF only). [file gh-19-1-1251-s2.pdf]

TABLE S2 Risk of Cardiovascular Outcomes for Time in Target Range of Resting Heart Rate in Sensitivity Analysis (AF only)

TIR	Model 1		Model 2		Model 3	
	HR (95% CI)	p Value	HR (95% CI)	p Value	HR (95% CI)	p Value
Major adverse cardiovascular events						
0%~25%	0.984(0.68,1.43)	0.934	0.99(0.68,1.44)	0.944	0.96(0.66,1.41)	0.843
>25%~50%	1.27(0.83,1.95)	0.263	1.23(0.80,1.88)	0.343	1.22(0.79,1.86)	0.369
>50%~100%	1.00(Reference)	…	1.00(Reference)	…	1.00(Reference)	…
Per 1 SD	1.03(0.87,1.21)	0.751	1.02(0.87,1.21)	0.798	1.03(0.87,1.22)	0.724
All-cause Mortality						
0%~25%	1.07(0.68,1.70)	0.766	1.10(0.69,1.75)	0.685	1.08(0.68,1.72)	0.735
>25%~50%	1.37(0.82,2.29)	0.233	1.31(0.78,2.19)	0.314	1.29(0.77,2.17)	0.334
>50%~100%	1.00(Reference)	…	1.00(Reference)	…	1.00(Reference)	…
Per 1 SD	1.00(0.82,1.22)	0.991	0.99(0.81,1.21)	0.886	0.99(0.81,1.22)	0.949

HR = hazard ratio.

Model 1: adjusted for age, gender, minority, randomization to rate vs. rhythm control strategies and history of smoking;

Model 2: further adjusted for past medical history of hypertension, diabetes mellitus, coronary artery disease and heart failure status by NYHA class symptoms;

Model 3: further adjusted for history of coronary artery bypass graft, interventional procedure and pacemaker implantation
